# The Role of Selected Epigenetic Pathways in Cardiovascular Diseases as a Potential Therapeutic Target

**DOI:** 10.3390/ijms241813723

**Published:** 2023-09-06

**Authors:** Anna Wołowiec, Łukasz Wołowiec, Grzegorz Grześk, Albert Jaśniak, Joanna Osiak, Jakub Husejko, Mariusz Kozakiewicz

**Affiliations:** 1 Department of Geriatrics, Division of Biochemistry and Biogerontology, Collegium Medicum in Bydgoszcz, Nicolaus Copernicus University, 87-100 Torun, Poland; 2 Department of Cardiology and Clinical Pharmacology, Faculty of Health Sciences, Collegium Medicum in Bydgoszcz, Nicolaus Copernicus University, 87-100 Torun, Poland

**Keywords:** epigenetics, cardiovascular diseases, heart failure, ten-eleven translocation enzymes, TET enzymes

## Abstract

Epigenetics is a rapidly developing science that has gained a lot of interest in recent years due to the correlation between characteristic epigenetic marks and cardiovascular diseases (CVDs). Epigenetic modifications contribute to a change in gene expression while maintaining the DNA sequence. The analysis of these modifications provides a thorough insight into the cardiovascular system from its development to its further functioning. Epigenetics is strongly influenced by environmental factors, including known cardiovascular risk factors such as smoking, obesity, and low physical activity. Similarly, conditions affecting the local microenvironment of cells, such as chronic inflammation, worsen the prognosis in cardiovascular diseases and additionally induce further epigenetic modifications leading to the consolidation of unfavorable cardiovascular changes. A deeper understanding of epigenetics may provide an answer to the continuing strong clinical impact of cardiovascular diseases by improving diagnostic capabilities, personalized medical approaches and the development of targeted therapeutic interventions. The aim of the study was to present selected epigenetic pathways, their significance in cardiovascular diseases, and their potential as a therapeutic target in specific medical conditions.

## 1. Introduction

Epigenetics involves a diverse set of reversible modifications to the genome that do not alter the deoxyribonucleic acid (DNA) sequence. Both the external environment and the internal microenvironment of cells and tissues influence epigenetic mechanisms that play a key role in the specification of cell fate and development of the organism [1,2]. Growing evidence suggests that epigenetic activity plays a fundamental role in the regulation of pathophysiological cellular processes [3,4,5]. The involvement of epigenetic mechanisms has been demonstrated at the level of protein and ribonucleic acid (RNA) gene expression and DNA replication in repair processes, cell differentiation and embryogenesis [6]. Epigenetic marks have traditionally been considered immutable, potentially transmissible to progeny, and underlying stable differentiation into different cell types that express their own distinctive patterns of gene expression [7]. It has now become clear that chromatin epigenetic markers are dynamically regulated in response to environmental cues [8,9,10]. This has led to a shift in the use of epigenetics to account for transient changes in chromatin in response to external stimuli that control gene expression [11,12,13,14,15]. Epigenetic pathways can be divided into three main categories: DNA methylation, histone modifications, and non-coding RNAs [16]. The first two are referred to as direct epigenetic mechanisms due to their involvement in chromatin remodeling, while non-coding RNAs are considered to be indirect due to modulation of gene expression mainly in the post-transcriptional phase. 

### 1.1. DNA Methylation

DNA methylation is mainly associated with the inhibition of gene expression [17]. Active genes are located primarily in unmethylated sites. Cytosines contained in the DNA chain undergo covalent methylation in a reaction catalyzed by DNA methyltransferases (DNMT). High levels of 5-methylcytosine (5-mC) formed in this way are characteristic of the inactive form of chromatin-heterochromatin. The 5-mC molecule acts similarly to ordinary cytosine (C) by combining with guanine (G) in double-stranded DNA. However, when methylated cytosines are present at 5′–C–phosphate–G–3′ (CpG) sites in the promoter and enhancer regions, genes are often repressed [18,19]. CpG islands are regions in the genome with an increased content of CpG dinucleotides compared to the average value for the entire genome [20]. In humans, the presence of CpG islands within the promoter affects as much as 60–70% of genes. CpG islands located near housekeeping genes are usually not methylated. In the case of genes whose expression is characteristic only for specific tissues, methylation occurs selectively—CpG islands are not methylated only in the cells of these specific tissues; in others, they are methylated and not expressed [21].

More than every fifth transcription factor is inhibited from binding when the recognition sequence contains 5-mC. In addition, the presence of 5-mC in the promoter region can attract methyl-CpG-binding domain proteins that interact with nucleosome remodeling and histone deacetylase, leading to gene silencing. There are two types of DNA methylation—maintenance and de novo. Maintenance methylation is responsible for the methylation of the newly synthesized DNA strand due to replication at sites complementary to the methylated sites in the parent strand. This allows the methylation pattern to be inherited after cell division. DNMT-1 is involved in this reaction, responsible for maintaining epigenetic markers, which allows them to be reproduced with each cell division. The second type of methylation—de novo—uses DNMT-3A and DNMT-3B and consists of attaching methyl groups in completely new places, which changes the methylation pattern [22]. This allows it to maintain a specific pattern of methylated DNA sequences for various organs and tissues, which is passed on to the daughter cell. Thanks to this, it knows which genes should be expressed. Additionally, transcription from methylated CpG islands is strongly and hereditarily suppressed [22]. The reverse process—demethylations of 5-mC—catalyze ten-eleven translocation (TET) enzymes [23].

In eukaryotes, DNA is packaged into repeating units called nucleosomes by wrapping multimeric histone proteins. When nucleosomes are organized in tightly packed bundles (heterochromatin), transcription is inhibited by blocking the access of the transcriptional machinery. Conversely, when the chromatin is relaxed (euchromatin), the nucleosomes resemble balls on a string and this condition is associated with active transcription [24]. A large part of the modifications within histones relates to the change in the affinity of histones for DNA, which translates into the state of chromatin order. Although histone modifications occur throughout the sequence, the unstructured N-termini of histones, called histone tails, are particularly heavily modified [25]. Among the many modifications that occur, such as acetylation, methylation, ubiquitination, phosphorylation, sumoylation, ribosylation, and citrullination, the best described mechanism is histone acetylation. Histone acetylation involves the attachment of an acetyl group to a lysine (K) residue at the N-terminus of the core histone. These ends form protruding tails, and their acetylation reduces the affinity of histones for DNA, loosening the chromatin structure. In heterochromatin, histones are mostly non-acetylated. Histone acetyltransferases (HATs) are involved in acetylation. Similarly, histone methylation and demethylation are achieved by histone methyltransferases and histone demethylases, respectively [26]. Histone methylation can induce both activation and repression of transcription, depending on the number and location of the methyl groups [27,28]. Research on the diversity of histone modifications and the interactions resulting from them led to the formulation of the histone code hypothesis. According to the hypothesis, specific histone modification patterns determine the state of chromatin and gene activity in its area, which causes a specific biological effect. However, it is now known that the function of many histone-modifying enzymes extends independently and goes beyond their catalytic activity [29]. A full understanding of the exact mechanisms by which these histone tail changes affect DNA–histone interactions remains in the realm of research, though some specific examples have been developed in detail.

### 1.2. Indirect Epigenetic Mechanisms

**Figure 1:**
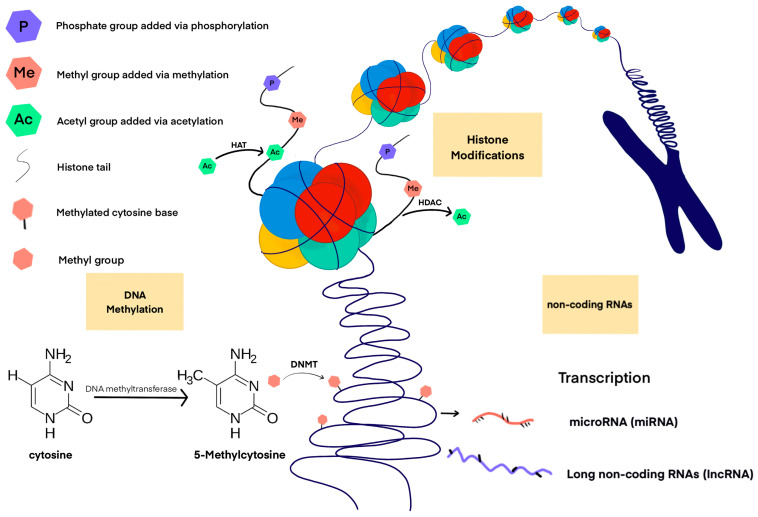
Major epigenetic mechanisms involved in CVDs.

A non-coding RNA (ncRNA) is a functional RNA molecule that is not translated into a protein. Particularly noteworthy in the context of epigenetic machinery are long ncRNAs (lncRNAs), and small RNAs, which include, among others: microRNAs (miRNAs), small interfering RNAs (siRNAs) and piwi-interacting RNAs (piRNAs). Approximately 60% of human protein-coding genes appear to be regulated by miRNAs [30]. MiRNA binds to messenger RNA (mRNA) that is complementary to it, leading to gene silencing and blocking the translation of mRNA into protein. As post-transcriptional repressors, they act on the 3′ untranslated region of mRNA to induce its degradation or translational repression. Each miRNA expressed in a cell can target about 100 to 200 messenger RNAs that it downregulates [31]. At the same time, about 50% of miRNA genes are associated with CpG islands [32], therefore, the expression of these genes is dependent on DNA methylation. Other miRNAs are regulated epigenetically by histone modifications or by combined DNA methylation and histone modification [32]. lncRNAs in the vast majority of cases do not encode any protein, there is evidence that some of them can carry information about micro- and polypeptides [33,34]. Specific lncRNAs show different subcellular locations—most of these molecules can be found in the nucleus, less in the cytoplasm or in both locations simultaneously. As a result, lncRNA molecules can efficiently regulate gene expression at various levels, from epigenetic DNA modifications and transcriptional changes to modulation of mRNA stability and translational or post-translational control [35]. In addition, lncRNAs can act as competitive endogenous RNAs (ceRNAs), thereby blocking miRNAs and regulating epigenetic mechanisms [36]. Figure 1 shows major epigenetic mechanisms involved in CVDs.

### 1.3. Epigenome-Wide Association Study

An epigenome-wide association study (EWAS) is a study that analyzes a set of measurable epigenetic characteristics such as DNA methylation across a genome. It is conducted in different patients to establish associations between epigenetic variation and a specific identifiable feature that can be considered within particular cardiovascular and cardiometabolic diseases [8]. An important meta-analysis of a genome-wide association study is a paper published in late 2022 in Nature, which covered *n* = 115,150 cases of all-cause heart failure and *n* = 1,550,331 controls of diverse genetic background [37]. The researchers identified 47 heart failure (HF) risk loci. Colocalization, gene expression profiling and Mendelian randomization have provided consistent evidence for a special role for branched-chain keto acid dehydrogenase E1 subunit alpha (BCKDHA) in the development of HF from all causes. In total, proteome-wide Mendelian randomization identified nine circulating HF-related proteins, and the strongest evidence of colocalization involved nine genes- DNAJC18, MTSS1, SQLE, BCKDHA, ABO, ALPK3, and PROM1. Another study demonstrated the utility of methyl-binding domain-capture sequencing to evaluate peripheral blood DNA methylation markers in a cohort of cardiac surgical patients with severe multi-vessel coronary artery disease and phenotypic extremes of heart failure [38]. DNA methylation patterns from peripheral blood allowed to distinguish patients with coronary artery disease with and without HF. The analysis of the methylation of CpG sites during the first diagnosis and follow-up enables the identification of new prognostic biomarkers and genes involved in the pathomechanism of CVDs. This approach was used in a paper from this year that shows significant upregulation of SOCS3, ITGAL, NFIC, NCOR2, and PGK1 mRNA (qRT-PCR) in peripheral blood mononuclear cells from pulmonary arterial hypertension (PAH) patients compared to healthy controls (*p* ≤ 0.05) [39]. This is an important study that shows new therapeutic and prognostic hopes in the care of patients with a poor prognosis. Further promising clinical implications, from two studies published in 2023, identify new hypermethylated or hypomethylated CpG and gene sites associated with anthracycline-induced cardiomyopathy [40,41]. Researchers indicate that the differential expression of epigenetic modifiers in early and late cardiotoxic heart failure reveals DNA methylation as a key regulator of cardiotoxicity during anthracycline treatment [41]. Since anthracycline-induced cardiomyopathy is still the leading cause of late morbidity in childhood cancer survivors [40], these epigenetic findings hold great potential for clinical applications. Further research in this area may contribute to reducing the cardiotoxicity of oncological treatment, which should become the subject of clinical trials. The variability of methyl tags of selected genes and miRNAs among HF patients is summarized in Table 1.

EWAS provides broad insights into the DNA methylation landscape of heart tissue among patients and confirms the role of DNA methylation in regulating genes involved in the progression of CVDs. The data collected with EWAS not only indicate new targets for potential epidrugs, but also provide the opportunity to conduct additional functional analysis of the role of identified methylation-sensitive genes in order to determine their exact mechanistic role in the development of CVDs. Further exploration of cell type-specific methylation patterns of the epigenetic machinery will provide valuable insights into the involvement of individual cells (cardiomyocytes, fibroblasts, endothelial cells, inflammatory cells) in the progression of CVDs that either drive or result from CVDs pathogenesis. This proposed analysis would indeed explain the differential cellular composition that can be seen in some CVD pathologies [42].

## 2. The Role of Epigenetics in Inflammation

Much evidence indicates that epigenetic mechanisms mediate the development of inflammation by modulating the expression of tumor necrosis factor alpha (TNF-α), interleukin 1 (IL-1), interleukin 2 (IL-2), interleukin 6 (IL-6), interleukin 8 (IL-8), interleukin 12 (IL-12), granulocyte-macrophage colony-stimulating factor (GM-CSF), tumor suppressor genes, oncogenes and autocrine and paracrine activation of the nuclear factor kappa-light-chain-enhancer of activated B cells (NF-κB) transcription factor [43,44,45,46,47,48,49,50]. It is believed that cardiac stress, e.g., during myocardial infarction (MI), leads to NF-κB-dependent stimulation of pro-inflammatory cytokine gene expression in resident heart cells [51,52,53]. The release of these mediators, as well as the molecular patterns associated with damage, induces the recruitment of inflammatory cells such as neutrophils and monocytes to the myocardium. This condition may have a beneficial dimension, e.g., by promoting the phagocytic removal of dead cells after an infarction [54,55]. On the other hand, persistent extravasation of inflammatory cells may lead to inflammation of the heart with its subsequent remodeling [56,57]. Inflammation-induced vascular change pathways result in both hypertrophic and hypertensive responses. Many of them are caused by the activation of the transcription factor NFκB, which ultimately leads to changes in gene transcription and the perpetuation of a hypertensive state [58,59].

Epigenetic regulation of both local and high-order chromatin structure is one of the mechanisms involved in the development of HF. Recently, early events of adaptive chromatin remodeling have been shown to precede pathological phenotypes and are reinforced in the failing heart [60]. Myocardial stress gene transcription responds through the dynamics of H3K27 acetylation enhancer enrichment and co-regulatory gene network [61]. Changes in gene expression in the course of HF correlated with differential enrichment of H3K27 histone acetylation in their respective proximal and distal interacting genomic enhancers encapsulated in these static chromatin structures [61]. Researchers indicate that a robust and intact CCCTC-binding factor loop is required to elicit a rapid and accurate stress response [61]. In order for transcription factors to activate their target genes, DNA and chromatin must be remodeled, mainly due to the aforementioned modifications within histones by histone acetyltransferase (HAT) and histone deacetylase (HDAC), which either open or close DNA strands to transcription factors. Normally, this balance is tightly controlled, but under conditions of stress and inflammation, activation of pro-inflammatory cytokines can result in increased HDAC activation and histone acetylation, which correlates with an increase in NFκB activity and a further increase in pro-inflammatory cytokine expression [62]. Due to the plasticity of epigenetic changes and their readiness to respond to the intervention of small molecule inhibitors, there is great potential for the development of new therapeutic agents that will serve as direct or complementary therapeutic compounds [63,64]. HDAC inhibitors have shown great potential in inhibiting the development of inflammatory diseases [65,66] and play a clear immunomodulatory role in the heart [67,68] reducing cardiac dysfunction associated with its aging [69]. In addition, HDAC inhibitors reduce myocardial fibrosis by multiple mechanisms including inhibition of proliferation and/or migration of cardiac fibroblasts and induction of genes that suppress extracellular matrix production by fibroblasts [65]. They also inhibit proinflammatory signals of fibrosis, differentiation of monocyte precursors into mature collagen-producing fibrocytes, and endothelial–mesenchymal transition (EndoMT), which defines the process of pathological dedifferentiation of vascular endothelial cells into matrix-producing mesenchymal cells [65].

Since inflammation has long been recognized as an important component of the pathogenesis of heart failure (HF) [58,59,70,71], the therapeutic efficacy of HDAC inhibitors has been investigated in animal models of HF. Numerous studies have shown that HDAC inhibitors are effective therapeutic agents that can halt and reverse the remodeling and dysfunction of the heart associated with the development of CVDs [72]. One study involved spontaneously hypertensive rats treated for 20 weeks with valproic acid (VPA), a weak HDAC inhibitor. Researchers observed reductions in the expression of IL-1β and TNF-α in the left ventricle, which showed a correlation with attenuated myocardial hypertrophy and fibrosis, and improved cardiac function [73]. Another pan-HDAC inhibitor, suberoylanilide hydroxamic acid (SAHA), approved by the Food and Drug Administration (FDA) for the treatment of cutaneous T-cell lymphoma, decreased plasma concentrations of more than 20 inflammatory cytokines including IL-1α, IL2, and TNF-α, inflammatory cell infiltration, interstitial collagen deposition, systolic hypertension and myocardial interstitial fibrosis in a model of hypertension-induced cardiac remodeling [74]. Another pan-HDAC inhibitor, trichostatin A (TSA), has shown the ability to inhibit NOS2 expression caused by various toll-like receptors (TLR) activators by lipopolysaccharides (LPS) or *Escherichia coli*, which are activators of TLR4 signaling, double-stranded RNA, which is an activator of TLR3 signaling, or peptidoglycan, which is an activator of TLR2 signaling. TSA led to increased acetylation of mitogen-activated protein kinase (MAPK) phosphatase-1, thereby promoting its ability to inhibit pro-inflammatory p38 kinase signaling. TSA also inhibited the expression of TNF-α, IL-6 and IL-1β in LPS-stimulated macrophages [75]. In addition, HDAC-mediated deacetylation of the regulatory factor interferon-7 has been shown to be required for effective DNA binding of this proinflammatory transcription factor [76]. Finally, the protein65 (p65) subunit of NF-κB can be deacetylated by HDAC3, promoting its nuclear shutdown by binding to nuclear factor of kappa light polypeptide gene enhancer in B-cells inhibitor, alpha (IκBα) [77].

Genome-wide association studies have revealed that genetic variants at the locus corresponding to HDAC-9 are associated with the risk of stroke, coronary artery disease and atherosclerosis due to varying levels of HDAC-9 expression [78,79,80]. HDAC-9 has been shown to play an essential role in cholesterol homeostasis and inflammation, as deletion of HDAC-9 in systemic cells and bone marrow resulted in upregulation of lipid homeostasis genes, downregulation of inflammatory genes and polarization of macrophages towards the M2 phenotype through increased accumulation total acetylated H3 and H3K9 on the ATP-binding cassette transporter (ABCA-1) and ATP-binding cassette subfamily G member 1 (ABCG-1) promoters in macrophages [80]. Researchers believe that targeted inhibition of HDAC9 may be a viable strategy to prevent atherosclerosis [81].

The presence of inflammatory activated macrophages in the aortic wall is a pathological feature of abdominal aortic aneurysm (AAA) development [82]. Thanks to the characterization of surgical samples and studies on animal models, the role of epigenetic modifications in monocytes and macrophages formed from them in the pathogenesis of aortic aneurysms has been demonstrated [7,83]. Global methylation levels in peripheral blood mononuclear cells are significantly altered in CpG islands in AAA patients compared to controls and positively correlated with increased aortic diameter [84]. Human AAA samples showed between five- and fifteen-fold higher levels of HDAC1, 2, 4, and 7 mRNA compared to healthy controls, while no differences were observed for HDAC3, 5, and 8 [85]. In mouse models of AAA, treatment with class I HDAC inhibitors (MS-275) or class IIa (MC-1568) reduced the incidence of AAA, the inflammatory phenotype of macrophages, and the concentration of pro-inflammatory mediators [85]. Another epigenetic therapeutic target for the treatment of AAA may be microRNA-33. MiR-33-deficient bone marrow transplantation reduced AAA formation in a mouse model by reducing inflammation by several mechanisms, including reducing matrix metalloproteinase-9 in macrophages and monocyte chemotactic protein-1 in vascular smooth muscle cells [86].

Other important molecules include the bromodomain and extra terminal (BET)-containing protein family. BET proteins act as an epigenetic reader that is a key positive regulator of NF-κB and transforming growth factor TGF-β dependent pro-inflammatory gene expression [87], acting as a critical co-activator in the process of cardiomyocyte hypertrophy [88]. Bromodomain-containing protein 4 (BRD4) binds acetyl-lysine via bromodomains and co-activates transcription, forming complexes that signal to RNA polymerase II and react directly with NF-κB through acetylated Lys310 of the p65 subunit. This interaction is required for NF-κB transactivation [89]. Treatment with the BET inhibitor thienodiazepine (JQ1) was shown to have a therapeutic effect during severe HF induced by prolonged pressure overload as well as massive anterior MI in a mouse model, and early intervention with JQ1 at the very onset of pressure overload in mice prevented the development of cardiac hypertrophy and left ventricular (LV) systolic dysfunction. JQ1 attenuated many of the hallmarks of HF progression in vivo, including cardiomegaly, pulmonary edema, LV systolic dysfunction, LV cavity dilation, LV wall thickening, and LV fibrosis [88]. Because BRD4is involved in the maintenance and progression of cancer cells in a wide range of malignancies [90]. BRD inhibitors, including JQ1 derivatives, are the subject of human therapy studies in early clinical phases [91,92]. Unlike several cancer drugs that cause cardiotoxicity, researchers indicate that BET bromodomain inhibitors, such as HDACs, may be a privileged class of cancer drugs that have cardioprotective properties due to the regulation of pro-inflammatory genes [88].

Another FDA-approved targeting molecule for BDR4 is apabetalone (APA)—it affects several characteristics of the atherosclerotic process, such as lipid metabolism, oxidative stress and vasculitis. Adding APA therapy to patients receiving high-dose statins led to an increase in high-density lipoprotein cholesterol (HDL-C) by 15.4% and a decrease in high-sensitivity C-reactive protein (hsCRP) by 18.4% after 24 weeks of treatment, which was a better result than the placebo group. Improvement in the lipid profile was associated with fewer cardiovascular events in patients treated with APA than with placebo [93]. In pooled phase 2 data analyses, APA reduced the rate of cardiovascular events by approximately 60%. However, in the BETOnMACE Phase III study, APA reduced the risk of major adverse cardiac events (MACE) by 50% in the chronic kidney disease subpopulation, indicating a beneficial effect along the kidney–heart axis [94,95]. Certainly, further clinical trials are needed to better investigate the safety and efficacy of APA in the treatment of CVDs. An overview of studies on the use of HDCAC inhibitors in CVDs is summarized in Table 2. An overview of clinical trials of both new epidrug molecules and already commonly used drugs due to their additional epigenetic mechanisms is summarized in Table 3.

Many drugs using epigenetic mechanisms of gene silencing have long been used in the treatment of humans, mainly for oncological causes. These include DNMT inhibitors, such as 5-azacytidine and 5-aza-2′-deoxycytidine, or HDAC inhibitors, such as vorinostat and romidepsin. Numerous more modern drugs from these groups (belinostat, panobinostat, etinostat, SAHA, givinostat, guadecitabine) are currently used as part of standard oncological treatment and clinical trials [109]. Non-oncology therapies include, but are not limited to, rheumatic diseases (systemic onset juvenile idiopathic arthritis—givinostat) or neurological diseases (amyotrophic lateral sclerosis—sodium phenylbutyrate–taurursodiol) [110,111,112]. Drugs commonly used in the treatment of CVDs, such as statins, sodium–glucose cotransporter-2 inhibitors (SGLT2i), hydralazine or metformin, in which the pleiotropic effect has been observed for a long time, have recently been identified as drugs showing an epigenetic mechanism [113]. Metformin, by activating AMPK, consequently leads to the modification of histones and promotes an increase or decrease in the expression of several genes. Metformin also indirectly increases HAT1 activity as observed in a mouse embryonic fibroblast model [114]. Metformin treatment also reduced the activity of two important co-activators of a large number of genes involved in inflammation and gluconeogenesis, HAT p300 and CREB-binding protein [114,115]. Both phenomena are strongly represented in patients with HF [113]. Moreover, long-term exposure to metformin was associated with a protective effect on the incidence of newly diagnosed symptomatic heart failure with preserved ejection fraction (HFpEF), diastolic dysfunction and LV hypertrophy in patients with type 2 diabetes and hypertension, which may be beneficial in delaying the progression of HFpEF [116]. Hydralazine, a vasodilator, exerts epigenetic effects by reducing the expression of DNMT1. In addition, this drug can modulate calcium homeostasis in cardiomyocytes by reducing methylation of the SERCA2a promoter, while enhancing the protein and activity of SERCA2a [117]. The epigenetic effects of this drug are also linked to specific gene expression by methylation of CpG islands in gene promoters [113]. Going forward, dapagliflozin (SGLT2i) modulates miRNAs involved in the pathophysiology of HF, such as miR199a-3p and miR30e-5p, which are involved in the regulation of PPARδ levels of mitochondrial fatty acid oxidation [113,118].

Statins are drugs that regulate epigenetic mechanisms at all levels—DNA methylation, histone modification and via non-coding RNAs [113]. They normalize subtelomeric DNA methylation in type 2 diabetes patients [119] and induce epigenetic changes by modulating Sirt1 transcription, resulting in regulation of inflammatory and apoptotic mechanisms. Statins increase miRNA-221/222 levels, the downregulation of which is associated with coronary artery disease [120] while upregulated miR-22 levels are associated with myocardial hypertrophy [121]. Statin therapy, by restoring KLF4-miR-483 expression and inhibiting EndoMT, may benefit patients with acute Kawasaki disease [122]. However, a pilot study showed that statins differentially modulate microRNA expression in the peripheral cells of patients with hyperlipidemia, which may be related to the variable response to these drugs [123].

## 3. DNA Methylation and Its Importance in the Regulation of the Cardiovascular System

**Figure 2:**
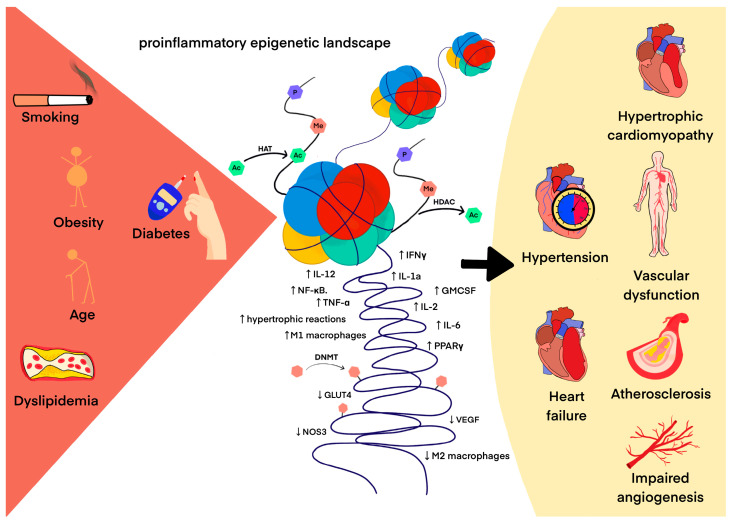
The pro-inflammatory epigenetic landscape as a result of adverse environmental interactions in the development of CVDs. The arrow indicates cause and effect.

The best-known epigenetic modification of DNA is 5-cytosine methylation, which is essential for proper gene expression, transposon silencing, alternative splicing and genome stability [124]. Cardiovascular risk factors such as smoking, low dietary folic acid, and elevated plasma homocysteine induce an abnormal DNA methylation pattern that may determine an individual’s predisposition to develop CVDs [8,125]. The methylation status of hundreds of CpG sites has been used as an epigenetic clock to measure the biological age that determines the rate of human aging [126,127]. In 832 participants without CVDs, researchers determined the difference between chronological and biological age using DNA methylation analysis and the Havroth formula. There were 153 cases of CVDs during the 10-year follow-up. For each year of increased biological age, the researchers observed a 4% increase in CVD risk. (HR = 1.033, 95% CI 1.004–1.063, *p* = 0.024) [128]. Since abnormal DNA methylation patterns occur before macrovascular damage [129], their identification may improve individual cardiovascular risk stratification. A 2023 screening study analyzed various methylated genes using a methylated DNA immunoprecipitation chip (MeDIP-chip). The study revealed that hypomethylation within the vascular endothelial growth factor B (VEGF-B), placental growth factor (PLGF), phospholipase C beta1 (PLCB1) and fatty acid transporter 4 (FATP4) genes could represent new biomarkers for CVDs. In addition, the VEGF-receptor signaling pathway regulated by DNA methylation may play a role in the pathogenesis of cardiovascular diseases in diabetes [130]. Moreover, global DNA hypomethylation is observed in atherosclerotic lesions both in humans and in animal models [131,132], whereas the promoter regions of atherosclerotic protective genes such as estrogen receptor β, ABCA1 and Krüppel-like factor 4 (KLF4) are often hypermethylated in atherosclerosis [133]. To date, the mechanisms underlying changes in DNA methylation in cardiovascular aging and CVDs remain under investigation. Several observations documented so far relate, for example, to the correlation between increased DNMT1 in macrophages and decreased PPAR-γ and increased pro-inflammatory cytokines in apolipoprotein E (ApoE)-deficient mice fed an atherogenic diet as well as in patients with atherosclerosis [134]. Another study describes hypomethylated CpG in the endothelial nitric oxide synthase (eNOS) promoter of eNOS-expressing endothelial cells, in stark contrast to dense DNA methylation in eNOS-negative cells (e.g., vascular smooth muscle cells) [135]. DNMT1 has been shown to be a critical regulator that negatively regulates arterial stiffening by maintaining the vascular contractility phenotype of smooth muscle cells [136], and mitochondrial DNA hypermethylation in vascular smooth muscle cells impairs cell contractility [137]. Methylome profiling allows a thorough insight into the pathomechanism of individual diseases, reports from 2023 reveal the key electrophysiology and immune dysregulation in hypertrophic cardiomyopathy, which brings new therapeutic possibilities [138]. DNA methylation also has an important aspect in radiation-induced cardiovascular disease (RICVD), which is an important problem in thoracic radiotherapy with complex pathophysiology. Current studies describe a systemic radiation fingerprint at the level of DNA methylation, explaining the possible relationship of DNA methylation to the pathophysiology of RICVD [139]. Figure 2 illustrates a cause-and-effect diagram between the drivers of proinflammatory epigenetic changes that contribute to the development of CVDs.

## 4. The Role of Tet Enzymes in Active DNA Demethylation and Its Relationship with the Differentiation and Function of Cardiovascular Cells

Members of the methylcytosine dioxygenase family, Tet enzymes regulate transcription by playing a major role in DNA demethylation by catalyzing the initial hydroxylation of 5-mC to 5-hmC. All three related human proteins, Tet1, Tet2 and Tet3, possess 5-mC oxidase activity but differ in domain architecture and tissue specificity of their expression levels [140]. The basic characteristics are summarized in Table 4.

The 5-hmC and 5-mC profiles mapped in embryonic, neonatal, adult, and hypertrophic mouse and human cardiomyocytes indicate dynamic modulation of DNA methylation and hydroxymethylation during development and heart disease [141]. Deposition of 5-hmC on the gene body is strongly positively correlated with gene expression and identifies heart-specific genes. Disruption of these processes contributes to the phenotypic expression of CHDs such as tetralogy of Fallot (TOF) or ventricular septal defect (VSD) [142]. Genome-wide methylation analysis shows that Tet knockout (Tet-KD) causes hypermethylation of the promoter of genes encoding WNT inhibitors, leading to hyperactivated WNT signaling and defects in cardiac mesoderm patterning. Human embryonic stem cells in which all three Tet genes were inactivated had defective structural gene specifications for cardiac progenitor cells and formed into cardiomyocytes with altered mesodermal patterning. Among other factors, this was due to the inability to maintain the Tet-dependent hypomethylation state and the expression of the NKX2-5 gene [141]. In another study, Tet2/3 double-KD mice display an embryonic lethal ventricular non-compaction cardiomyopathy [143]. Recent studies of DNA methylation in biopsies of human heart tissue from patients with chronic heart failure (CHF) of various etiologies have shown clear epigenomic patterns in important DNA elements of the heart genome in end-stage HF [142,144]. Interestingly, DNA methylation patterns in CVDs share some common features with developmental changes. Methylation patterns of cardiomyocytes isolated from failing mouse hearts partly resemble those of newborn mice [145]. While DNA methylation in failing cardiomyocytes returned to the fetal methylation pattern, the observed changes were less pronounced during physiological myocyte aging. Research is currently lacking to determine whether human cardiomyocyte DNA methylome exhibits similar characteristics during development and disease. The 5-hmC landscape is also shifting towards a neonatal distribution pattern in pathologically hypertrophied hearts. The RNA-seq data revealed that all three Tet genes were highly expressed in the fetal cardiomyocyte genome and downregulated in adult cells, and undergoing some changes during hypertrophy induction [146]. The most strongly expressed member was Tet2, positively correlated with the expression of the absolute 5-hmC content. These findings led the researchers to hypothesize that Tet2 plays the largest role in the fetal cardiomyocyte genome. Researchers showed that Tet2 regulates the expression of key cardiac genes, such as the Myh7 fetal cardiac α-myosin heavy chain gene, by depositing 5-hmC on the gene body and within enhancers. Tet2-KD resulted in significant deregulation of a large number of genes including those related to the cell cycle, cardiac development and contraction of the heart muscle. The researchers emphasize that Myh7 and the myosin light chain gene Myl4—which encodes the basic structural elements of the embryonic heart muscle—were profoundly affected by Tet2-KD, going through six and three down adjustments, respectively. Although Tet2-KD significantly reduced the endogenous expression of Tet2 (Western blotting confirmed a 70% reduction of the Tet2 protein), the researchers found no effect of Tet2-KD on the global level of 5-hmC, which they explain by compensation by Tet3, whose level was significantly increased after Tet2-KD. This relationship emphasizes the high importance of the Tet family in the processes of differentiation of cells of the cardiovascular system. It has recently been discovered that dysmetabolic conditions such as diabetes mellitus can alter DNA demethylation in conjunction with extranuclear Tet relocation, which is dependent on AMPK activity [147]. It is currently unknown whether extranuclear Tet compartmentalization occurs in the heart of diabetic patients and whether this phenomenon plays a role in this context. In contrast, a study from 2022 with *n* = 150 patients with AMI [148] showed a significant increase in Tet-2 levels, positive correlation of Tet2 with infarct size, cTNT levels and Gensini score (all, *p* < 0.001). The researchers included the representation of people with CV-risk factors, including diabetes, in the control and study groups, noting the lowest Tet2 levels in the group with the lowest CV risk. In the same year, facilitated expression of Tet2 by c-MYC binding to the miR-29a-3p promoter was shown to have a therapeutic effect on ventricular remodeling in MI rats [149]. An increase in Tet2 improved cardiac function, reduced infarct size, myocardial apoptotic death, reduced oxidative stress, inflammatory response, and normalized collagen deposition. There are also preliminary reports on the use of Tet1 overexpression in controlling arrhythmias dependent on the secretory activity of cancer cells [150]. Researchers have linked gastrointestinal cancer secretions to DNA methylation of potassium voltage-gated channel subfamily D member 3 (KCND3) ion channel genes as a result of activation of transforming growth factor β/phosphoinositide 3-kinase (TGF-β/PI3K) signaling. Tet1 overexpression normalized the methylation state of the CpG islands of the promoters of the KCND3 channel genes in human cardiomyocytes, which protected the ion currents from the influence of tumor secretory activity [150]. Making changes in Tet expression certainly has a wide therapeutic potential and is currently an attractive subject of numerous scientific studies.

## 5. Conclusions

Epigenetics provides a thorough insight into the cardiovascular system, from its development to its continued functioning. The evidence cited suggests that epigenetics is a rapidly evolving science that may provide solutions to the continuing strong clinical impact of CVDs. This is possible by targeting pharmaceutical interventions to reprogram the pro-inflammatory epigenetic landscape associated with cardiometabolic and vascular diseases and to improve individual risk stratification.

## Figures and Tables

**Table 1:** Methylation of selected protein-coding genes and miRNAs in HF patients. Tabulated data come from analyses that reached statistical significance of *p* < 0.05.

Gene/miRNA	Direction of Methylation	Etiology of Heart Failure	Reference
**HEY2, MSR1**	Hypermethylated	HOCM	Nadezhda Glezeva et al. 2017 [42]
**COX17, MYOM3, miR24-1**	Hypermethylated	ISCM	
**CTGF, miR155**	Hypomethylated	ISCM	
**CTGF, MMP2**	Hypomethylated	DCM	
**SLC18A2/VMAT2, PDXK, SPTBN4,**	Hypermethylated	anthracycline-induced CM	Purnima Singh et al. 2023 [40]
**NIPAL2/SLC57A4, OR4D10, FARP1/PLE**	Hypomethylated	anthracycline-induced CM	
**HDAC9, JARID2, GREM1**	Hypomethylated	CAD	Bain CR et al. 2020 [38]
**PDSS2**	Hypermethylated	CAD	
**SOCS3**	Hypomethylated	PAH	Giuditta Benincasa et al. 2023 [39]

Abbreviations: CM: cardiomyopathy; DCM: dilated cardiomyopathy; HOCM: hypertrophic obstructive cardiomyopathy; ISCM: ischemic cardiomyopathy; PAH: pulmonary arterial hypertension; CAD: coronary artery disease; HEY2: hairy/enhancer-of-split related with YRPW motif protein 2; MSR1: macrophage scavenger receptor 1; COX17: cytochrome C oxidase copper chaperone; MYOM3: myomesin 3; CTGF: connective tissue growth factor; MMP2: matrix metallopeptidase 2; SLC18A2/VMAT2: solute carrier family 18 member A2/vesicular monoamine transporter 2; PDXK: pyridoxal kinase; SPTBN4: spectrin beta non-erythrocytic 4; NIPAL2/SLC57A4: nipa-like domain containing 2/SLC57A4; OR4D10: olfactory receptor family 4 subfamily D member 10; FARP1/PLE: rhoGEF and pleckstrin domain-containing protein 1/PLE; HDAC9: histone deacetylase 9; JARID2: jumonji and at-rich interaction domain containing 2; GREM1: Gremlin 1; PDSS2: decaprenyl-diphosphate synthase subunit 2; SOCS3: suppressor of cytokine signaling 3.

**Table 2:** An overview of studies on the use of HDCAC inhibitors in CVDs.

HDAC Inhibitor	Class Inhibition	Description of the Study	Cardiovascular Effect	Inflammation
RGFP966	III	an animal model of diabetic cardiomyopathy [96]	↓LVID, LV mass ↑EF, FS	↑IRS1, Akt phosphorylation, GLUT ↓PAI-1 TNF-α, lipid peroxides, ROS, fibrosis
VPA	I	an animal model of hypertension [73] an animal model of hypertension [97]	↓MAP, LVPWT ↓SBP, BW	↓IL-1β, IL-6 NFκ, ROS, fibrosis ↓triglycerides
SAHA	Pan-HDAC inhibitor	an animal model of hypertension [74] an animal model of a myocardial infarction [98]	↑ papillary muscle repolarization time and action potential time, ↓LV hypertrophy, SBP ↓ infarct size, cardiomyocyte death by 40%, ↑ systolic function	↓IL1a, CINC2a/b, IL17, MIP1a, IP10, IL2, TNFα, IL4, IL8, MIP1a, IFNγ, IL13, IL1b, CINC3, IL1ra, CNTF, IL3, GMCSF, IL6, RANTES, thymus chemokine, TIMP1, LIX, fractalkine, L-selectin, MIG, sICAM, IL10, MIP3, fibrosis ↑flow and autophagic activity in the borderline infarction zone
MS-275 MC-1568	I IIa	an animal model of a AAA [85]	↑survival, incidence and severity of AAA ↓aneurysm expansion assessed by Doppler ultrasonography	↓MCP-1,COX-2, IL-1β, IL-6
Tubastatin A	IIa	an animal model of cardiac hypertrophy and fibrosis in response to chronic angiotensin II signaling [99]	↑LVEF	↓CD45+, fibrocytes, transition from LC3-I to LC3-II
ITF2357 (givinostat)	Pan-HDAC inhibitor	an animal model of aging-related cardiac dysfunction with preserved EF [100]	↓diastolic dysfunction, LV wall thickness	↓fibrosis

Abbreviations: AAA: abdominal aortic aneurysms; EF: ejection fraction; LVID: left ventricular internal dimension; LV: left ventricle; FS: fractional shortening; IRS1: insulin receptor substrate 1; Akt: protein kinase B; GLUT4: glucose transporter type 4; PAI-1: plasminogen activator inhibitor 1; TNF-α: tumor necrosis factor alpha; ROS: reactive oxygen species; MAP: mean arterial pressure; LVPWT: left ventricular posterior wall thickness; IL: interleukin; NFκ: nuclear factor kappa-light-chain-enhancer of activated B cells; SBP: systolic blood pressure; BW: body weight; CINC: cytokine-induced neutrophil chemoattractants; MIP1a: macrophage inflammatory proteins 1a; IP10: interferon-inducible protein; IFNγ: interferon gamma CNTF: ciliary neurotrophic Factor, GMCSF: granulocyte macrophage-colony stimulating factor; TIMP1: metallopeptidase inhibitor 1 LIX: LPS-induced CXC chemokine; MIG: monokine induced by IFN-gamma; sICAM: soluble intercellular adhesion molecule, COX-2: cyclooxygenase-2 LVEF: left ventricular ejection fraction LC: light chain; ↑: upregulation; ↓: downregulation

**Table 3:** Clinical trials on the use of drugs in cardiovascular diseases.

Molecule Name	Mechanism of Action	Cardiovascular Disease	Clinical Trial Phase
**direct-acting epidrugs**			
Inclisiran	Synthetic siRNA; ↓hepatocyte PCSK9 expression	heterozygous familial hypercholesterolemia	III [101]
		elevated LDL-C despite maximum tolerated dose of LDL-C lowering therapies	
		atherosclerotic cardiovascular disease	
CDR132L	↓miR-132	heart failure	Ib [102]
		aschemic cardiomyopathy	
Apabetalone	↑ApoA-I gene expression by interacting with BRD4	acute coronary syndrome, diabetes	III [93]
		atherosclerosis	IIb [103]
**indirect acting epidrugs**			
Hydralazine	↓DNMT1 expression modulation of calcium homeostasis in cardiomyocytes by↓ SERCA2a methylation	HFpEF	II [104]
Metformin	Phosphorylation and ↓of epigenetic enzymes—HATs, class II HDACs, and DNMTs as a consequence of ↑AMPK	Pulmonary hypertension, HFpEF	II [105]
Dapagliflozin	AGE/RAGE signaling	HFpEF	III [106]
Empagliflozin, Dapagliflozin, Canagliflozin	JunD/PPAR-γ pathway	diabetic cardiomyopathy among HTX recipients	completed, observational clinical trial [107]
Rosuvastatin	↑miRNA-221/222, ↑KLF4-miR-483	ischemic HFrEF	III [108]

Abbreviations: PCSK9: proprotein convertase subtilisin/kexin 9; LDL-C: low-density lipoprotein cholesterol; ApoA-I: apolipoprotein AI; BRD4: bromodomain containing 4; DNMT1: DNA methyltransferase 1; SERCA2a: sarcoplasmic/endoplasmic reticulum Ca^2+^ ATPase 2a; HFpEF: heart failure with preserved ejection fraction; HATs: histone acetyltransferases; HDACs: histone deacetylases; DNMTs: DNA methyltransferases; AMPK: 5′AMP-activated protein kinase; AGE/RAGE: advanced glycation end products/receptor for advanced glycation end products; PPAR-γ: peroxisome proliferator—activated receptor gamma; HTX: heart transplantation; KLF4: Krüppel-like factor 4; ↑: upregulation; ↓: downregulation

**Table 4:** The basic characteristics of Tet enzymes.

Gene	Binding of the CpG Sequence	Converting 5-mC to 5-hmC	Occurrence
Tet1	the N-terminal CXXC zinc-finger domain	CD domain	Embryonic stem cells, primordial germ cells
Tet2	Lack of CXXC CXXC is represented by gene adjacent to Tet2 called Idax/Cxxc4, which cooperates with Tet2 negatively regulating its activity	CD domain	Embryonic stem cells, primordial germ cells
Tet3	Two isoforms are present: CXXC(+) and CXXC(-)	CD domain	Oocytes, zygotes

## Data Availability

Not applicable.
